# AFFECTIVE REACTIVITY TO DAILY STRESSORS AND IMMUNE GENE EXPRESSION IN THE MIDUS STUDY

**DOI:** 10.1093/geroni/igac059.1635

**Published:** 2022-12-20

**Authors:** Abner Apsley, Sun Ah Lee, Aarti Bhat, David Almeida, Idan Shalev

**Affiliations:** Pennsylvania State University, University Park, Pennsylvania, United States; The Pennsylvania State University, University Park, Pennsylvania, United States; The Pennsylvania State University, State College, Pennsylvania, United States; The Pennsylvania State University, University Park, Pennsylvania, United States; The Pennsylvania State University, University Park, Pennsylvania, United States

## Abstract

Past research indicates that an individual’s affective reactivity to daily stressors is associated with elevated levels of inflammatory biomarkers such as interleukin-6 and C-reactive protein. Here, we assessed individuals’ positive and negative affective reactivity to daily stressors, and their association with expression levels of genes in the conserved transcriptional response to adversity (CTRA; proinflammatory and type-1 interferon antiviral genes). We hypothesize that gene expression levels of CTRA genes are a possible explanatory mechanistic link between higher levels of affective reactivity and inflammatory markers. Our study included 199 individuals from the Midlife in the United States Refresher study (ages 26-71, 52.8% female, and 17.9% non-white) who participated in the Gene Expression and Daily Diary Projects. Individuals provided whole blood for gene expression analysis and completed an 8-day telephone interview to assess daily experiences. Positive and negative affective reactivity scores were used to indicate magnitude of an individual’s changes in daily affect on stressor-days versus non-stressor-days, and were computed using multilevel modeling. Three CTRA gene expression scores were created for each individual, representing the average expression of proinflammatory, type-1 interferon, and a combination of both. Preliminary findings suggest a negative association between the CTRA composite score and negative affective reactivity to daily stressors (β = -0.963, p = 0.034), controlling for covariates. Further analyses will use affective reactivity to predict individual CTRA genes, correcting for multiple testing. This study will be the first to examine the relationship between daily assessments of emotions and gene expression levels in a representative U.S. cohort.

